# Robotic rehabilitation with the Lokomat^®^ system in the improvement of executive functions and processing speed in stroke patients: a quasi-experimental study

**DOI:** 10.3389/fnagi.2026.1795203

**Published:** 2026-04-09

**Authors:** Marina Esther Cabrera-Brito, María del Carmen Carcelén-Fraile, Agustín Aibar-Almazán, María Auxiliadora Márquez-Apolinario, Yolanda Castellote-Caballero

**Affiliations:** 1 Department of Health Sciences, Faculty of Health Sciences, University of Jaén, Jaén, Spain; 2 Island Rehabilitation Institute, S.L., Las Palmas de Gran Canaria, Spain; 3 Department of Educational Sciences, Faculty of Social Sciences, University of Atlántico Medio, Las Palmas de Gran Canaria, Spain; 4 International Scientific Association on Innovation in Education and Health (ACIINES), Jaén, Spain; 5 Department of Health Sciences, University of Fernando Pessoa-Canarias, Las Palmas de Gran Canaria, Spain

**Keywords:** executive functions, neuroplasticity, processing speed, robotic rehabilitation, stroke

## Abstract

**Objective:**

To evaluate the effects of robotic rehabilitation with the Lokomat^®^ system on executive functions and processing speed in stroke patients.

**Methods:**

A quasi-experimental pretest-posttest study was conducted with a sample of 136 patients with a confirmed stroke diagnosis. Participants were assigned to a robotic rehabilitation program using the Lokomat^®^ for 10 weeks, with 1-h sessions twice a week. Executive functions were assessed using the Trail Making Test (TMTA and TMTB), and cognitive processing speed was assessed using the Symbol Digit Modalities Test (SDMT) before and after the intervention. The Wilcoxon signed-rank test was used to compare the differences between pre- and post-intervention measurements.

**Results:**

The results showed significant improvements in TMTA, TMTB, and SDMT scores after the intervention (*p* < 0.001). Analysis of the mean difference revealed a notable improvement in executive functions and processing speed, with large effect sizes (Cohen’s d = −1.93 for TMTA, −2.08 for TMTB, and −1.27 for SDMT).

**Conclusion:**

Robotic rehabilitation with the Lokomat^®^ significantly improves executive functions and cognitive processing speed in stroke patients, suggesting its potential as a therapeutic tool in post-stroke neurological rehabilitation.

## Introduction

Stroke, also known as cerebrovascular accident, is one of the leading causes of disability worldwide. According to the World Health Organization (WHO), approximately 15 million people suffer a stroke each year, of whom more than 5 million are left with permanent impairments (Feigin et al., 2025). The consequences of stroke are diverse and affect both mobility and cognitive abilities. Motor impairments include paralysis, muscle weakness, and postural control difficulties, while cognitive impairments can involve deficits in memory, attention, information processing, and executive functions (Li et al., 2024). These impairments have a profound impact on patients’ quality of life, limiting their autonomy and their ability to perform activities of daily living (ADLs) (Ai et al., 2024). Post-stroke neurological rehabilitation plays a crucial role in functional recovery, helping patients regain independence and improve their overall well-being (Bisevac et al., 2022).

For decades, conventional rehabilitation has been the cornerstone of post-stroke treatment, encompassing physical and occupational therapies that focus primarily on restoring motor control and balance (Li et al., 2024). These interventions, while effective in many cases, have limitations, as they depend on the patient’s active participation and do not always induce significant improvements in cognitive functions, which are also affected by stroke (Kreiger et al., 2025). Furthermore, the conventional rehabilitation process can be slow and requires high patient motivation, which can lead to frustration, especially in those with severe sequelae (Shen et al., 2025; Yoshida et al., 2021).

In recent years, robotics has emerged as one of the most innovative technologies in the field of rehabilitation, offering new opportunities to optimize the recovery process (Banyai and Brișan, 2024). Robotic rehabilitation systems, allow for the intensive repetition of specific movements, essential for neurological rehabilitation, through the use of exoskeletons that assist lower limb movement (Tugonza, 2025). One of the most widely used devices for this purpose is the Lokomat^®^ system (Hocoma AG, Switzerland). This device combines a robotic orthosis with a treadmill and a partial body weight support (BWS) system, providing a controlled interface that allows for the repetition of physiological gait patterns in a safe and controlled environment (Diego et al., 2024). The use of Lokomat^®^ has proven effective in improving gait, balance, and functional independence in stroke patients through repetitive stimulation and the promotion of neuroplasticity, a key process in post-stroke rehabilitation (Baronchelli et al., 2021; Wu et al., 2023).

However, advances in robotic rehabilitation are not limited solely to motor improvement. In recent years, studies have begun to explore the impact of these technologies on cognitive functions, especially in key areas such as processing speed and executive functions (Aprile et al., 2020; Alam et al., 2025). Processing speed refers to an individual’s ability to efficiently receive, interpret, and respond to information. After a stroke, many patients experience a reduction in the speed at which they process cognitive signals, affecting their ability to perform everyday tasks quickly and effectively (Elendu et al., 2023; Su et al., 2015). In this regard, robotic rehabilitation, by providing repetitive and controlled training, could help activate neural networks related to attention, perception, and motor response key areas for improving information processing speed (Nizamis et al., 2021).

On the other hand, executive functions, which include skills such as planning, decision-making, inhibitory control, and cognitive flexibility, are also commonly affected after a stroke (Sakai et al., 2024b). These functions are essential for performing complex daily tasks and adapting to new situations. Robotic rehabilitation offers continuous and targeted stimulation that, by integrating motor and cognitive tasks, could induce improvements in these processes. In this context, recent studies have highlighted the relevance of cognitive–motor interference and dual-task rehabilitation approaches, which simultaneously engage motor execution and cognitive processing during gait training. These approaches aim to replicate real-world situations in which individuals must manage motor and cognitive demands at the same time, potentially enhancing both motor and cognitive recovery. Emerging research in robotic-assisted rehabilitation has shown promising results when combining robotic gait training with cognitive challenges, suggesting that such dual-task paradigms may further stimulate neuroplastic mechanisms and improve functional outcomes in neurological populations (Moshayedi et al., 2023; Moshayedi et al., 2026). The repetition of movements in a controlled environment could promote the reorganization of neural networks responsible for decision-making and planning, supporting the recovery of executive functions (Chen et al., 2025).

Neuroplasticity, a key mechanism in post-stroke rehabilitation, not only facilitates the recovery of motor functions but can also induce improvements in cognitive abilities (Aderinto et al., 2023). Robotic rehabilitation has the potential to activate brain areas related to both motor control and cognitive processing, promoting the reorganization of the neural networks that control both aspects (Chen et al., 2025). However, despite these advances, evidence on the effects of robotic rehabilitation on executive functions and processing speed in stroke patients remains limited. Therefore, it is crucial to further investigate how these technologies can improve not only motor function but also cognitive aspects, as both are essential for comprehensive recovery (Wang and Liu, 2025; Aprile et al., 2025).

For this reason, the objective of this study is to evaluate the effects of a robotic rehabilitation program using the Lokomat^®^ system on improving cognitive functions, particularly processing speed and executive functions, in stroke patients. This study seeks to provide empirical evidence on the potential of robotic rehabilitation to induce changes in cognitive performance, contributing to the understanding of its role in the comprehensive rehabilitation of post-stroke patients.

## Methods

### Design and participants

This study was conducted using a quasi-experimental pretest-posttest design with a single group. This design allows for the evaluation of the effects of a specific intervention, in this case, robotic rehabilitation with the Lokomat^®^ device, on cognitive outcomes such as executive functions and processing speed, comparing the results obtained before and after the intervention. The study received approval from the Ethics Committee of the Universidad del Atlántico Medio on March 7, 2025 (approval code: CEI/05-018), and was registered on ClinicalTrials.gov before commencement. Before the intervention began, all participants (or their legal representatives, when necessary) signed an informed consent form. The procedures were carried out in accordance with ethical principles and confidentiality, as outlined in the Declaration of Helsinki.

To be selected, participants had to meet the following inclusion criteria: (i) Confirmed diagnosis of stroke; (ii) Age ≥ 18 years (no upper age limit); (iii) Sufficient cognitive ability to follow simple instructions (Mini-Mental State Examination ≥ 24); (iv) Medical and hemodynamic stability allowing participation in intensive physiotherapy sessions; (v) Signed informed consent by the patient or their legal guardian; and (vi) Patients in the subacute stage of stroke recovery after medical stabilization and referral to the rehabilitation service. Participants were excluded if they met any of the following exclusion criteria: (i) Diagnosis of another neurological condition (such as Parkinson’s disease, multiple sclerosis, spinal cord injury); (ii) Metallic implants or electronic devices incompatible with the use of the Lokomat^®^; (iii) Cardiovascular instability or medical contraindications to moderate physical activity; and (iv) Simultaneous participation in other robotic rehabilitation programs or clinical studies.

### Recruitment and sampling

The sample size calculation was performed using G*Power software (version 3.1.9.7) assuming a paired-samples comparison for pretest–posttest measurements, which corresponds to the statistical structure of the study design. A small effect size (Cohen’s d = 0.25) was selected as a conservative estimate according to conventional criteria described in the statistical literature, considering the exploratory nature of the study and the variability typically observed in rehabilitation outcomes among stroke patients. A significance level of *α* = 0.05 and a statistical power of 80% (1 − *β* = 0.80) were assumed under a two-tailed hypothesis. With these parameters, it was determined that 128 participants were required. To compensate for a potential dropout rate of 5% (approximately seven participants), the target sample size was increased to 135 participants. Ultimately, 136 participants were enrolled, all of whom completed the study (with no dropouts), exceeding the target sample size and ensuring the planned statistical power.

A consecutive sample of all adults with a confirmed diagnosis of stroke who were referred to and admitted to the hospital’s rehabilitation service during the study period was used. Potential participants were identified from daily admission lists, outpatient schedules, and referrals from the neurology/rehabilitation services of referring hospitals. Selection, according to pre-established eligibility criteria, was carried out by trained physicians using medical records and a standardized checklist. Eligible patients (or their legal representatives) were contacted personally, received written information about the study, and signed written informed consent before the initial assessment. Reasons for exclusion or refusal (such as additional neurological conditions, contraindications for the use of the Lokomat^®^, cardiovascular instability, concurrent participation in robotic programs, or cognitive impairments that precluded eligibility) were documented in a selection record. To avoid selection bias, no socioeconomic or gender restrictions were imposed. Language requirements were limited to ensuring that participants could adequately understand the study procedures, which was verified through the cognitive eligibility criterion of a Mini-Mental State Examination (MMSE) score ≥24. Participants were recruited after the acute medical phase of stroke and were considered clinically stable to participate in the rehabilitation program. No financial incentives were offered. Sociodemographic data were collected to characterize the study sample, including age, sex, occupational status, educational level, use of pain medication, and the presence of sensory and motor symptoms. These variables were obtained from clinical records and patient interviews during the baseline assessment (Table 1).

**Table 1:** Baseline sociodemographic and clinical characteristics of the study sample.

(*n* = 136)		Total (*n* = 136)
Age		61.57 ± 1.11
Sex	Male	65 (47.80%)
	Female	71 (52.20%)
Occupational status	Retired	10 (7.4%)
	Worker	117 (86%)
	Unemployed	9 (6.6%)
Marital status	Divorced	47 (34.6%)
	Married	42 (30.9%)
	Single	47 (34.6%)
Educational status	Primary education	37 (27.2%)
	Secondary education	35 (25.7%)
	University education	40 (29.4%)
	No formal education	24 (17.6%)
Sensory symptoms	Yes	56 (41.2%)
	No	80 (58.8%)
Motor symptoms	Yes	63 (46.3%)
	No	73 (53.7%)
Pain medication	Yes	74 (54.4%)
	No	62 (45.6%)

Quantitative variables are presented as the mean and standard deviation. Qualitative variables are presented as frequency and percentage.

### Outcomes

The effects of robotic intervention in stroke patients were evaluated using a series of standardized and clinically validated instruments, all highly sensitive to changes in neurological rehabilitation settings. Measurements were taken at two time points: before the intervention began and at the end of the robotic rehabilitation program. To reduce detection bias, assessments were performed by trained clinicians who followed standardized written instructions and scoring manuals specific to each instrument. The evaluators were independent from the therapists delivering the intervention and did not have access to participants’ previous test scores during the post-intervention assessment. Evaluators used the same versions of the instruments at both measurement points and were instructed to adhere to pre-established test scripts.

#### Executive functions

The Trail Making Test (TMT) (Reitan, 1958) is a tool used to assess cognitive functions, particularly those related to motor and visual skills, under timed conditions. This test is divided into two sections: Part A (TMT-A) and Part B (TMT-B). Part A is designed to measure an individual’s attention and psychomotor speed, and consists of a task in which the participant must connect numbered circles in sequence. Part B, on the other hand, demands greater cognitive flexibility, as the participant must alternate between connecting circles with numbers and letters, allowing for the assessment of executive function, specifically the ability to switch between different types of information. The time taken to complete each part of the test is used as an indicator of performance: a longer execution time is generally associated with lower cognitive efficiency. To reduce potential practice effects associated with repeated cognitive testing, alternative versions of the TMT were administered during the post-intervention assessment whenever available.

#### Processing speed

The Symbol Digit Modalities Test (SDMT) (Smith, 2002) is a test used to measure cognitive processing speed. In this task, the participant must associate symbols with corresponding numbers according to a predefined code, all within a set time limit. The test consists of a series of symbols to which the corresponding number must be quickly assigned, and the task is performed under a time limit (90 s). Scores on this test are based on the number of correct responses the participant gives within the allotted time. A higher score indicates better processing speed, while a lower score may reflect difficulties in this cognitive area. To minimize familiarity with the stimuli and reduce potential learning effects between assessments, an alternative version of the SDMT was used during the post-intervention evaluation.

### Intervention

The intervention consisted of a robotic rehabilitation program using the Lokomat^®^ system (Hocoma AG, Switzerland), implemented as a structured motor–cognitive training program designed to provide intensive gait training while simultaneously engaging cognitive processes such as attention, executive control, and information processing during walking tasks. This device combines an automated robotic lower limb orthosis with a treadmill and a partial BWS system, allowing for the safe and repeatable reproduction of the physiological gait pattern. The Lokomat^®^ sessions were integrated into a rehabilitation plan that also included conventional physical therapy and/or occupational therapy, as clinically indicated.

Each session began with patient preparation, which included the individualized placement and adjustment of the suspension harness, followed by the attachment of the robotic orthosis to the hips, knees, and ankles. Special attention was paid to biomechanical alignment in the frontal and sagittal planes, adjusting segment lengths, pivot points, and joint centering to minimize postural compensations and ensure proper gait kinematics. Key training parameters were then calibrated: (i) Treadmill speed was adjusted between 0.8 and 2.5 km/h, increasing or decreasing in 0.1–0.3 km/h increments based on gait pattern stability (no knee collapse at stance or foot drag during swing), gait quality as assessed by the therapist (symmetry, step length, cadence), and physiological tolerance. If pattern loss, pain, or marked spasticity was observed, the speed was reduced and/or a short break was given. (ii) Tolerance was monitored every 5–10 min using the rating of perceived exertion scale (target Borg CR10 3–5), heart rate within a safe range defined by the clinician (approximately ≤60–70% of the age-predicted maximal heart rate), which was estimated using the conventional formula (220 − age). Heart rate was continuously monitored during training using a wearable heart rate sensor integrated into the rehabilitation monitoring system, and observation of fatigue/compensations. Progress was defined as meeting the following criteria during two consecutive sessions: gait guided correctly by the device without manual assistance, absence of knee collapse or foot drag, and a Borg CR10 ≤ 5 with stable vital signs. When these criteria were met, speed was increased by 0.1–0.3 km/h and/or the BWS was reduced. (iii) BWS is the percentage of body weight unloaded by the harness, initially prescribed between 40 and 60%, adjusted to ensure safe knee extension in stance and adequate foot separation. The BWS was reduced by 5–10% when the patient maintained, for ≥2 min, (a) knee stability in stance without manual assistance, (b) symmetry in step length within ~10%, and (c) no increase in compensatory trunk movements. The ultimate goal was to achieve ≤20% BWS, where possible, or maintain the highest functionally safe level. (iv) The level of robotic assistance (guidance force) ranged from 0 to 100%, according to the standard operating parameters of the Lokomat^®^ system. At the beginning of training, guidance force was typically set at high levels (approximately 80–100%) to provide greater movement assistance and ensure correct gait pattern execution. As patients demonstrated improved voluntary motor control and gait stability, the level of guidance force was progressively reduced to encourage active participation. (v) Range of motion (ROM) and guidance force were adjusted to minimize compensations, allowing for patient-initiated movement.

The training performed during each session was structured into three sequential phases, adapted to the patient’s functional level. These phases were implemented within each training session and allowed the therapists to progressively adjust the level of robotic assistance and task complexity according to the patient’s tolerance and motor performance: (i) Safe gait phase: In this first phase, the Lokomat^®^ fully guides the patient’s movement, requiring no voluntary muscle activation. It is primarily used for initial neuromuscular adaptation, learning the motor pattern, and familiarization with the robotic environment. (ii) Progressive physiological gait phase: The level of robotic assistance and weight support is gradually reduced, with the aim of promoting greater active patient participation, increasing voluntary motor control, and strengthening functional muscle activation. Specific tasks that challenge gait stability and rhythm are incorporated, such as exercises focused on step length symmetry, cadence regulation, controlled weight shifting between lower limbs, and maintaining rhythmic stepping patterns during treadmill walking. (iii) Functional task-oriented gait phase (visual biofeedback, therapeutic games, dual tasks): In the final phase of each session, patients train their gait with real-time visual biofeedback and therapeutic games focused on symmetry in step length, regularity, and weight shifting. Examples include goal-directed steps, symmetrical step length tracing, cadence/rhythm synchronization, and obstacle avoidance routes. Dual cognitive tasks (such as counting backward by threes, alternating letter and number sequences, and naming categories) and motor tasks (such as reacting to visual cues while walking) are incorporated to challenge attentional control and executive functions during gait. These dual-task activities followed a standardized protocol and were applied consistently across participants, with predefined categories of cognitive tasks implemented by the therapists to ensure uniformity in the intervention. Progression was achieved by increasing treadmill speed, reducing the BWS, increasing game difficulty (smaller targets/faster cues), or increasing the complexity of the dual tasks. When available, the FreeD^®^ Pelvic Module (Lokomat^®^ extension) was activated to enable controlled pelvic movement in the lateral and transverse planes, facilitating physiological center-of-mass displacement and improving dynamic postural control during gait training. Additionally, the FreeD^®^ module, an extension of the Lokomat^®^, was used to enable active pelvic mobilization in the lateral and transverse planes, simulating physiological center-of-mass displacement. This function promotes training in dynamic postural control, trunk activation, and balance stabilization during the gait cycle.

Participants enrolled in a structured robotic gait training program using the Lokomat^®^ system. The intervention lasted 10 weeks and consisted of two training sessions per week, each lasting approximately 60 min. The following description refers to the procedures applied during each training session of the robotic rehabilitation program. Each session included approximately 5 min for preparation and warm-up, 40–45 min of robotic gait training (including the task-oriented phase), and 5–10 min for cool-down. Short standing breaks (30–120 s) were allowed when necessary to maintain gait pattern quality and ensure patient safety. To reduce performance bias, the same treatment protocol and progression criteria were applied to all participants, and therapists followed standardized operating procedures for device setup, parameter adjustment, and safety monitoring. Lokomat^®^ training was integrated into the participants’ ongoing multidisciplinary rehabilitation program. To facilitate functional transfer, robotic gait training was coordinated with conventional therapist-led sessions focused on balance training, transfers, and ADLs. The clinical rehabilitation team aligned session goals (e.g., weight shift, stride length symmetry, and cadence) so that conventional therapy could reinforce the motor patterns practiced with the device. In addition to the Lokomat^®^ sessions, participants continued to receive routine multidisciplinary rehabilitation (e.g., conventional physical and/or occupational therapy) according to the institution’s standard clinical protocols, supervised by the same rehabilitation team throughout the study period.

The protocol was applied in an individualized and progressive manner, dynamically adjusting the parameters according to the patient’s functional progress. This approach allowed working under the principles of intensity, repetition, specificity, and motor variability, which are essential to induce neuroplasticity, improve motor learning, and facilitate the functional transfer of gait to the real environment.

### Statistical analysis

In this study, which used a pretest-posttest design with a single group and no control group, the statistical analysis aimed to determine whether there was a significant difference between pre- and post-intervention measurements. The process began with a descriptive analysis of the data, which included calculating the measures of central tendency and dispersion (mean and standard deviation) of the variables at both measurement points. Subsequently, the Kolmogorov–Smirnov test was applied to verify the normality of the data. If the data followed a normal distribution, a paired-samples test was used. Otherwise, non-parametric tests such as the Wilcoxon signed-rank test were employed. In all analyses, a *p*-value <0.05 (two-tailed) was considered statistically significant. All analyses were performed using IBM SPSS Statistics (version 20). The statistical approach was predefined: *α* = 0.05 (two-tailed), reporting 95% confidence intervals and effect sizes for the change scores. Data distribution was assessed using the Kolmogorov–Smirnov test. When the assumption of normality was satisfied, a paired-samples *t*-test was used to compare pre- and post-intervention measurements. When the data did not meet the normality assumption, the non-parametric Wilcoxon signed-rank test was applied. A significance level of *p* < 0.05 (two-tailed) was adopted for all analyses.

## Results

A total of 136 participants completed the study, of whom 65 (47.80%) were women and 71 (52.20%) were men. The mean age of the participants was 61.57 ± 1.11 years, with an age range of 23 to 93 years.

### Executive functions

The TMTA variable, assessed using the Trail Making Test, showed a reduction in scores after the intervention. The mean score decreased from 80.87 ± 24.05 before the intervention to 72.75 ± 21.66 after the intervention.

Similarly, TMTB scores decreased following the intervention, with mean values changing from 137.20 ± 28.45 before the intervention to 123.51 ± 25.60 after the intervention.

The normality of both variables (TMTA and TMTB) was assessed using the Kolmogorov–Smirnov test, which indicated that the data did not follow a normal distribution (*p* < 0.001). Therefore, the Wilcoxon signed-rank test was used to compare pre- and post-intervention measurements. The results showed statistically significant improvements after the intervention (*p* < 0.001 for both variables).

**Figure 1:**
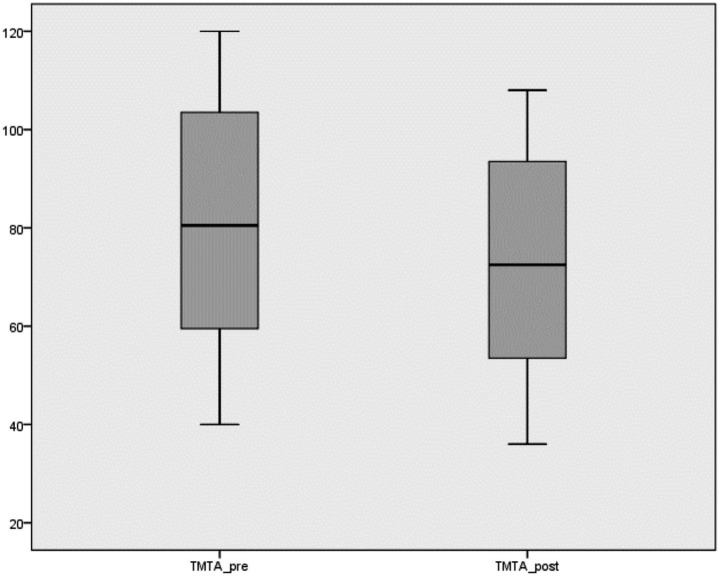
Boxplot showing the distribution of TMTA scores before and after the intervention.

Figure 1 illustrates the distribution of TMTA scores before and after the intervention.

**Figure 2:**
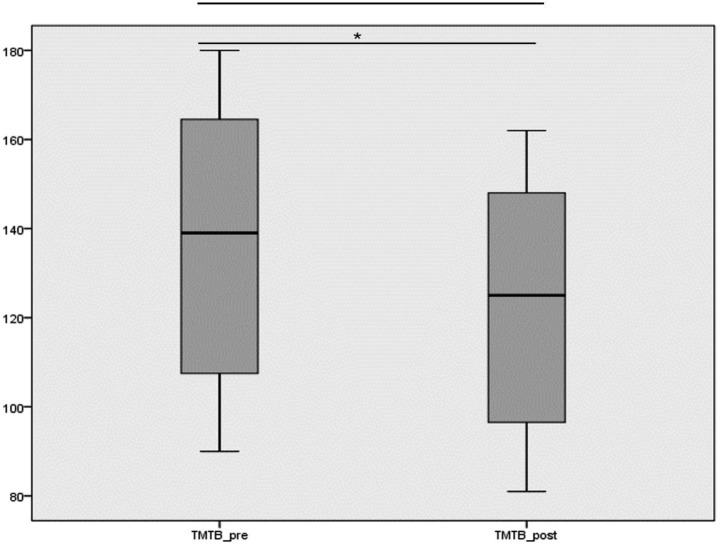
Boxplot showing the distribution of TMTB scores before and after the intervention. * Statistically significant difference between pre- and post-intervention measurements (Wilcoxon signed-rank test, *p* < 0.001).

Figure 2 shows the comparison of TMTB pre and TMTB post intervention scores. The boxplot reveals the distributions of scores before and after the intervention, highlighting the shift in the median and the overall decrease in the variability post-intervention.

Since the data did not follow a normal distribution, the Wilcoxon signed-rank test was used to analyze the differences between pre- and post-intervention measurements for the TMTA and TMTB variables. The analysis showed significant improvements after the intervention for both variables (*p* < 0.001). Specifically, the mean difference was −8.12 points for TMTA and −13.69 points for TMTB (Table 2), indicating improved executive function performance following the intervention.

**Table 2:** Results of the Wilcoxon signed-rank test for TMTA, TMTB, and SDMT.

Variable	Mean difference (pre – post)	*p*-value	Cohen’s *d* (effect size)	Interpretation
TMTA	−8.12 points	*p* < 0.001	−1.93	Significant improvement
TMTB	−13.69 points	*p* < 0.001	−2.08	Significant improvement
SDMT	−4.45 points	*p* < 0.001	−1.27	Significant improvement

Statistical significance was defined as *p* < 0.05. Cohen’s d values are interpreted as follows: small (0.2), medium (0.5), and large (0.8) effect sizes. TMT, trail making test; SDMT, symbol digit modalities test.

### Processing speed

**Figure 3:**
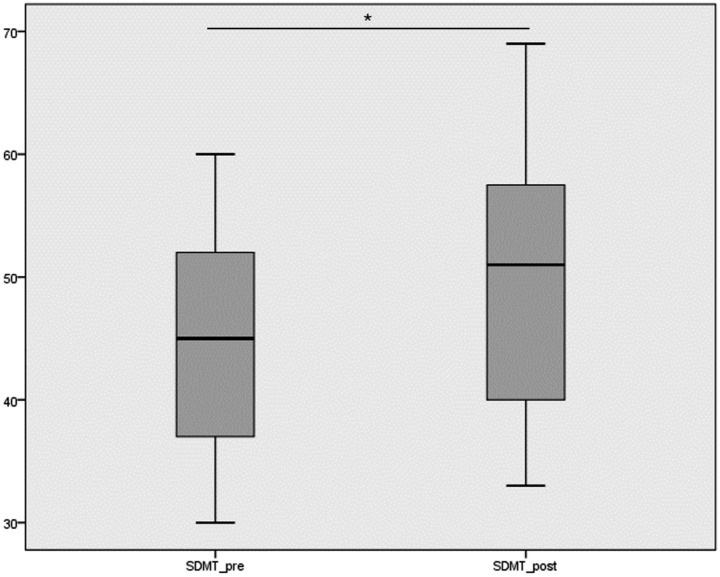
Boxplot showing the distribution of SDMT scores before and after the intervention. * Statistically significant difference between pre- and post-intervention measurements (Wilcoxon signed-rank test, *p* < 0.001).

The processing speed variable, assessed using the Symbol Digit Modalities Test (SDMT), showed a significant improvement after the intervention. Before the intervention, the mean score was 44.92 ± 8.71 points, while after the intervention the mean score increased to 49.37 ± 9.60 points. The normality of the SDMT variable was assessed using the Kolmogorov–Smirnov test, which indicated that the data did not follow a normal distribution (*p* < 0.05). Therefore, the Wilcoxon signed-rank test was used to compare pre- and post-intervention measurements. The results showed a statistically significant improvement after the intervention (mean difference = −4.45 points; *p* < 0.001), indicating better cognitive processing speed following the robotic rehabilitation program (Figure 3).

Since the data did not follow a normal distribution, the Wilcoxon signed-rank test was used to analyze the differences between pre- and post-intervention measurements of the SDMT variable. The test results were statistically significant (*p* < 0.001), indicating a significant difference in scores before and after the intervention. Specifically, the mean difference was −4.45 points (*p* < 0.001), suggesting a significant improvement in participants’ performance on the digit substitution task after the intervention (Table 2).

## Discussion

This study evaluated the effects of robotic rehabilitation with the Lokomat^®^ system on improving executive functions and cognitive processing speed in post-stroke patients, showing significant improvements in the measured outcomes following the intervention in both cognitive domains. Our results suggest that robotic rehabilitation may contribute to improvements in cognitive processes such as processing speed and executive functions, which are fundamental to the overall recovery of stroke patients.

Executive function deficits are common in post-stroke patients and are known for their negative impact on patients’ ability to perform daily life tasks. Executive functions encompass a range of cognitive skills essential for daily functioning, such as planning, decision-making, inhibitory control, and cognitive flexibility (Sakai et al., 2024a). These functions are necessary for managing complex situations, adapting to changes in the environment, and making informed decisions. In stroke patients, deficits in these areas can lead to difficulties completing everyday activities such as time management, organization, and problem-solving efficiently (Gibson et al., 2022).

The present study showed a significant improvement in the Trail Making Test (TMT) scores, both Part A and Part B, after robotic intervention. These two parts of the test assess attentional speed and cognitive flexibility, respectively. The reduction in execution times suggests greater cognitive efficiency, which may reflect improvements in executive functioning. This finding is consistent with previous research indicating that robotic rehabilitation, by providing a structured and repetitive environment for stimulation, may support the reorganization of neural networks associated with planning and cognitive control (Aprile et al., 2020; Xie et al., 2024; Bonanno et al., 2023).

A key aspect of post-stroke rehabilitation is neuroplasticity, which allows the brain to reorganize itself to recover lost motor and cognitive functions. Neuroplasticity is a fundamental process underlying functional recovery in stroke patients and can be modulated by various therapeutic interventions (Aprile et al., 2020). In this regard, robotic rehabilitation has been suggested to support changes in neural networks involved in cognition processes (Amirbekova et al., 2025; Moskiewicz and Sarzyńska-Długosz, 2025). The repetition of movements assisted by the Lokomat^®^ may contribute to the activation of both motor and cognitive networks, promoting brain reorganization. This process not only contributes to the recovery of movement but also facilitates the reactivation of neural circuits responsible for executive functions, improving key aspects of cognition such as decision-making, planning, and impulse control (Yuan et al., 2021; Lo et al., 2010).

It is important to highlight that the patient’s active participation in gait and balance tasks, combined with exercises that engage cognitive functions (such as the use of visual biofeedback and therapeutic games that challenge attention and planning), can induce synergistic improvements in cognitive processes (Ramos-Murguialday et al., 2013). The integration of motor and cognitive tasks within a single rehabilitation protocol could explain why patients improve in multiple areas simultaneously, as both brain networks (motor and cognitive) are activated and strengthened through repetition and conscious effort (Bonanno et al., 2023; Lee and Kim, 2025).

Furthermore, processing speed also improved significantly after the intervention. In this study, the improvement in the Symbol Digit Modalities Test suggests that repetitive, controlled training in a motor context may facilitate improvements in the speed at which patients process information. This improved processing speed allows patients to interact with their environment more efficiently and effectively, enhancing their ability to perform daily tasks more quickly and accurately (Wang et al., 2022). This finding reinforces the idea that combining motor training with cognitive tasks can activate brain regions associated with both motor execution and rapid information processing, as observed in previous research (Bertani et al., 2017; Tang et al., 2024).

Importantly, the interpretation of these findings must take into account that participants received concurrent conventional rehabilitation alongside robotic training. Conventional rehabilitation has been widely demonstrated to be effective in improving motor function, functional independence, and, to some extent, cognitive performance in stroke patients, particularly in domains such as attention and task-oriented functioning (e.g., occupational therapy interventions) (Bisevac et al., 2022; Gibson et al., 2022). However, previous literature suggests that the effects of conventional rehabilitation on higher-order cognitive domains, such as executive functions and processing speed, are often variable or limited, and may depend on the intensity and specificity of cognitive engagement during therapy (Wang and Liu, 2025; Chung et al., 2013).

The effect sizes observed in this study were notably large, particularly for executive function measures such as the TMT-B. While these values suggest substantial improvements following the intervention, they should be interpreted with caution. Given the quasi-experimental design and the absence of a control group, it is not possible to fully rule out the potential influence of other factors such as spontaneous recovery, concurrent rehabilitation therapies, or practice effects associated with repeated testing. Additionally, the combined nature of the intervention may have contributed to the magnitude of the observed effects. Therefore, although the findings suggest a positive association between robotic rehabilitation and improvements in cognitive performance, future randomized controlled studies will be necessary to more precisely determine the magnitude of the intervention’s effect.

In summary, robotic rehabilitation robotic rehabilitation may contribute to improvements in cognitive functions essential for daily life, such as executive functions and processing speed, within a multidisciplinary rehabilitation context (Hong et al., 2024). The repetitive and intensive stimulation of motor and cognitive neural networks through technologies like the Lokomat^®^ may promote neuroplastic mechanisms and stimulate neural networks associated with cognitive processing, contributing to the recovery process in post-stroke patients. These findings suggest that robotic rehabilitation may represent a valuable complementary tool in post-stroke rehabilitation programs for supporting cognitive recovery. However, improvements in motor outcomes such as gait and functional independence have been primarily reported in previous studies and were not directly evaluated in the present research (Zengin-Metli et al., 2018).

Although the results of this study are promising, it is important to acknowledge certain limitations. First, the quasi-experimental pretest-posttest design with a single group does not allow for definitive causal relationships to be established between the intervention and the observed improvements. Although the robotic intervention showed significant effects, the lack of a control group limits the generalizability of these findings. In future studies, it would be valuable to implement a randomized controlled trial (RCT) to confirm the efficacy of robotic rehabilitation in a larger and more diverse sample of stroke patients. Furthermore, the specific nature of the sample, which included only patients with stroke and some cognitive ability, may have influenced the results. It would be relevant to evaluate the impact of robotic rehabilitation in populations with acute stroke or greater cognitive impairment to determine its applicability in more heterogeneous contexts. Another aspect to consider is the duration and frequency of the intervention. Although 10 weeks of robotic rehabilitation twice a week produced significant improvements in cognitive and motor functions, it is unknown whether a longer duration or a more frequent protocol could further enhance these effects. Further research exploring different intervention parameters, such as session intensity and frequency, is needed to optimize robotic rehabilitation programs and establish best practices for their application in post-stroke rehabilitation.

## Conclusion

The findings of this study suggest that robotic rehabilitation using the Lokomat^®^ system may be associated with improvements in executive functions and processing speed in post-stroke patients. These results contribute to the growing body of evidence suggesting that robotic-assisted gait training may support both motor and cognitive recovery, potentially through neuroplastic mechanisms. However, the findings should be interpreted with caution due to the quasi-experimental design without a control group, which limits the ability to establish definitive causal relationships. Future studies using more robust designs, including randomized controlled trials, neuroimaging measurements, and long-term follow-up, are needed to better determine the specific contribution of robotic rehabilitation to cognitive recovery after stroke.

## Data Availability

Due to the sensitive nature of the information collected, participants were assured that their data would remain confidential and would not be publicly shared. Datasets generated and analyzed during the current study are available from the corresponding author upon reasonable request.
